# The application of epiphenotyping approaches to DNA methylation array studies of the human placenta

**DOI:** 10.21203/rs.3.rs-3069705/v1

**Published:** 2023-06-26

**Authors:** Almas Khan, Amy M Inkster, Maria S Peñaherrera, Suzanne King, Sue Kildea, Tim F Oberlander, David M Olson, Cathy Vaillancourt, Ursula Brain, Ella O Beraldo, Alexander G Beristain, Vicki L Clifton, Giulia F Del Gobbo, Wan L Lam, Gerlinde AS Metz, Jane WY Ng, E Magda Price, Johanna M Schuetz, Victor Yuan, Élodie Portales-Casamar, Wendy P Robinson

**Affiliations:** BC Children’s Hospital Research Institute (BCCHR); BC Children’s Hospital Research Institute (BCCHR); BC Children’s Hospital Research Institute (BCCHR); McGill University; University of Queensland; BC Children’s Hospital Research Institute (BCCHR); University of Alberta; Centre Armand Frappier Santé Biotechnologie - INRS and University of Quebec Intersectorial Health Research Network; BC Children’s Hospital Research Institute (BCCHR); BC Children’s Hospital Research Institute (BCCHR); BC Children’s Hospital Research Institute (BCCHR); University of Queensland; BC Children’s Hospital Research Institute (BCCHR); British Columbia Cancer Research Centre; University of Lethbridge; University of Calgary; BC Children’s Hospital Research Institute (BCCHR); BC Children’s Hospital Research Institute (BCCHR); BC Children’s Hospital Research Institute (BCCHR); BC Children’s Hospital Research Institute (BCCHR); BC Children’s Hospital Research Institute (BCCHR)

**Keywords:** DNA methylation, placenta, epiphenotyping, epigenetics, epigenetic age, gestational age, cell composition, ancestry

## Abstract

**Background:**

Genome-wide DNA methylation (DNAme) profiling of the placenta with Illumina Infinium Methylation bead arrays is often used to explore the connections between *in utero* exposures, placental pathology, and fetal development. However, many technical and biological factors can lead to signals of DNAme variation between samples and between cohorts, and understafinding and accounting for these factors is essential to ensure meaningful and replicable data analysis. Recently, “epiphenotyping” approaches have been developed whereby DNAme data can be used to impute information about phenotypic variables such as gestational age, sex, cell composition, and ancestry. These epiphenotypes offer avenues to compare phenotypic data across cohorts, and to understand how phenotypic variables relate to DNAme variability. However, the relationships between placental epiphenotyping variables and other technical and biological variables, and their application to downstream epigenome analyses, have not been well studied.

**Results:**

Using DNAme data from 204 placentas across three cohorts, we applied the PlaNET R package to estimate epiphenotypes gestational age, ancestry, and cell composition in these samples. PlaNET ancestry estimates were highly correlated with independent polymorphic ancestry informative markers, and epigenetic gestational age, on average, was estimated within 4 days of reported gestational age, underscoring the accuracy of these tools. Cell composition estimates varied both within and between cohorts, but reassuringly were robust to placental processing time. Interestingly, the ratio of cytotrophoblast to syncytiotrophoblast proportion decreased with increasing gestational age, and differed slightly by both maternal ethnicity (lower in white vs. non-white) and genetic ancestry (lower in higher probability European ancestry). The cohort of origin and cytotrophoblast proportion were the largest drivers of DNAme variation in this dataset, based on their associations with the first principal component.

**Conclusions:**

This work confirms that cohort, array (technical) batch, cell type proportion, self-reported ethnicity, genetic ancestry, and biological sex are important variables to consider in any analyses of Illumina DNAme data. Further, we demonstrate that estimating epiphenotype variables from the DNAme data itself, when possible, provides both an independent check of clinically-obtained data and can provide a robust approach to compare variables across different datasets.

## Background

The placenta is essential for fetal growth and development, and plays an important role in mediating maternal exposures that may influence newborn and child health. To better understand these roles of the placenta, genome-wide DNA methylation (DNAme) profiling has been widely applied, often using Illumina Infinium Methylation bead arrays. Alterations in placental DNAme have been reported in association with maternal exposures such as smoking(1, 2), gestational diabetes, and obesity(3–5), as well as in association with perinatal complications such as preeclampsia, chorioamnionitis, and low birthweight(6–11). In some cases, these effects are intersectional: for example, smoking-associated changes in placental DNAme may be confounded, or in some cases mediated, by lower birth weight(1, 2, 12), although other lifestyle and exposure factors can complicate interpretation of these data. Despite the range of studies conducted in placenta, replication analyses of epigenome-wide association studies (EWAS) in independent populations are less common. Even in the context of early-onset pre-eclampsia, which is a condition associated with widespread alterations in DNAme of large effect size, reported findings are often inconsistent in independent datasets(7, 13, 14).

Part of the issue underlying incomplete replication between studies is inter-dataset heterogeneity. Prior to performing epigenome-wide analysis, it is important to understand and account for the factors driving variability in each DNAme dataset. Relevant technical factors may include differences in sample processing techniques, batch effects, and poor data quality control, which can all lead to false positive EWAS results, or low signal-to-noise ratios(6). Biological factors that may confound analyses include differences in bulk tissue sample cell composition(15), sex(16), and gestational or chronological age (17, 18). In addition, the ethnicity and/or genetic ancestry of subjects are known confounders in EWAS studies(19–21), and many regions of high DNAme variability across individuals are influenced by genetic variation(22, 23).

To improve replicability across studies from diverse populations and with different sample collection methods, “epiphenotyping” approaches have been developed whereby the (epigenetic) DNAme data are used to extract information about phenotypic variables, such as age or sex. In the context of placental DNAme studies, for example, cell composition can be estimated from bulk tissue DNAme data, and can be used to assess and account for variation between samples(15). Similarly, genetic ancestry of the placenta, which may not be well captured by self-reported parental ethnicity, can be estimated from placental DNAme data directly as a continuous variable, which can be accounted for to improve replication between EWAS studies(24). Gestational age at birth, which is sometimes missing or inaccurately recorded in clinical records, can also be estimated from placental DNAme profiles(18). Estimating epigenetic gestational age could have utility in many contexts, including providing researchers with a standardized measure of gestational age comparable across studies, and enabling studies of placental epigenetic age acceleration(18). Since the development of these tools and their implementation in the PlaNET R package (25), the interactions between these placental epiphenotyping variables, their associations with other technical and biological variables, and their application to EWAS have not been fully characterized.

In this study, we use three cohorts of placental samples to assess factors contributing to within- and between-cohort variation in placental DNAme data. We specifically apply the PlaNET R package to estimate gestational age, ancestry, and cell composition epiphenotype variables, and we evaluate the utility of these epiphenotyping approaches, assess inter-cohort differences, and examine their relationships to technical and biological variables. In addition, we explore how technical and biological variables common to placental DNAme studies are related to each other, and to the imputed epiphenotype variables. Finally, based on these extensive studies, we provide a set of recommendations for the use of these epiphenotyping tools in placental EWAS.

## Results

### Cohort characteristics

In this study, we use DNAme data from 204 placentas across three independent cohorts to investigate the relationships between placental epiphenotype variables computed with the R package PlaNET, and other technical and biological characteristics associated with these samples. The placentas were sourced from three cohorts consisting of: (i) V-NORM, a normative population of pregnancies recruited at BC Women’s Hospital (BCWH) in Vancouver, Canada (n = 35), as part of a study on Epigenetics in Pregnancy Complications (EPIC)(7, 26, 27); (ii) V-SSRI, a population of pregnant individuals recruited in Vancouver, Canada in the 20th week of gestation (n = 64), with/without clinical depression, and with/without the use of selective serotonin reuptake inhibitors (SSRIs) (28, 29); and (iii) QF2011, a population of pregnancies, with a wide range of exposure to sudden-onset stress during gestation due to catastrophic flooding in the Australian state of Queensland in early January 2011 (n = 105)(30).

The placentas from V-NORM and V-SSRI were obtained and sampled in one processing lab located in Vancouver, Canada. The QF2011 placentas were collected and sampled in Brisbane, Australia, and subsequently snap-frozen and shipped to Montreal, Canada for DNA extractions. The QF2011 DNA samples were shipped to Vancouver for DNAme array processing. Samples from all three cohorts were assayed on the Illumina Infinium MethylationEPIC (850K) array at one centre in Vancouver, Canada, and were randomized during array processing for key variables including cohort, SSRI and flood-related stress exposure, and infant sex. Exclusion criteria applied to all cohorts included pregnancies with multiple fetuses, and/or chromosome abnormalities. The V-SSRI and QF2011 cohorts were prospectively recruited cohorts, and except for four individuals giving birth between 35.7–37.0 weeks, all others occurred at term. Cases for V-NORM were accordingly selected to have similar gestational ages at birth (i.e. ≥ 36 weeks) to match the other two cohorts, with four samples included between 36–37 weeks gestational age. Additionally, only for V-SSRI, participants with bipolar illnesses, hypertension, current substance abuse, any diabetes, or infants with congenital brain malformations or fetal growth restriction were excluded. Pregnancies affected by preeclampsia were excluded from V-NORM. The respective exclusion criteria were applied to all cohorts to enable examination of key variables of interest without the presence of large confoufinding factors (such as chromosome abnormalities or preeclampsia being associated with DNAme alterations, or bipolar illness possibly confoufinding depression analyses in the V-SSRI study). Key technical and biological variables used in this study are reported in Table 1.

In addition to the analyses conducted on PlaNET epiphenotype variables, we note that although these three cohorts underwent similar sampling protocols and were processed for DNAme analysis at one centre, there are some key between-cohort demographic differences that we considered during analyses (Table 1). First, maternal self-reported ethnicity/race (see Methods) differed between cohorts (p < 0.001), with almost all mothers from the QF2011 cohort identifying as white. Infant birth weight standard deviation (corrected for sex and gestational age) was also slightly higher in the QF 2011 cohort (p = 0.04). Finally, sample processing and storage in the QF2011 cohort differed in subtle, but potentially significant ways (see Methods). These key differences could contribute to cohort differences that were important to consider in data analysis.

### Main drivers of DNAme variation across cohorts

**Figure 1:**
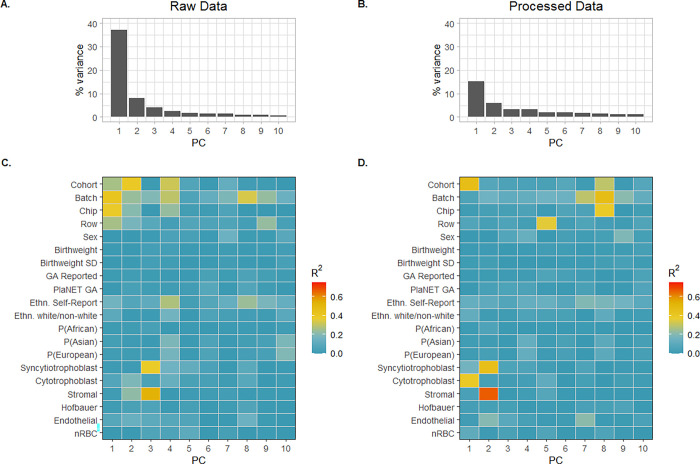
Principal Component Analysis of DNAme variation. Scree plots (A and B) show the proportion of variance explained by each principal component (PC), while heatmaps (C and D) show the R^2^ values of association from linear models run independently for each metadata variable (i.e., PC ~ Variable). (A) PC scree plot on raw data. (B) PC scree plot on processed, dasen + noob normalized data. (C) Raw data R^2^ heatmap showing strength of association between each PC from A and metadata variables. (D) Processed data R^2^ heatmap showing strength of association between each PC from B and metadata variables. For all plots, “SD” refers to standard deviation, “GA” refers to gestational age at birth, “Ethn” is used to denote ethnicity, P(African/Asian/European) represent the continuous probabilities from PlaNET ancestry prediction, and nRBC refers to nucleated red blood cells estimated by PlaNET.

Before performing DNAme array data analysis, it is useful to assess the main drivers of DNAme variation in the raw and processed data. To that end, we used principal component analysis (PCA) in combination with linear models to assess the relationship between variation in the data (PCs) with major technical and biological variables (Fig. 1).

Our first main finding was that data processing (normalization and probe filtering) attenuated the association between DNAme variance and technical factors, and the reduced DNAme variance in the processed data was instead related to cohort and cell-type differences rather than technical factors. This was evidenced by the fact that in the raw data, the first two principal components (PC1 and PC2) accounted for nearly half (45.2%) of the DNAme variability across all samples, and were associated with cohort (p < 0.001 for Cohort = V-NORM, V-SSRI, or QF2011) and technical array variables (batch, chip, row, all p < 0.05) (Fig. 1). After data processing, the proportion of DNAme variation explained by PC1 and PC2 decreased to 15% and 6%, respectively, and PC1 was no longer strongly associated with batch, chip, or row effects. In the processed data PC1 remained significantly associated with cohort (p < 0.001, R^2^ > 0.25) and cytotrophoblast proportion (p value < 0.001, R^2^ > 0.25), while PC2 was also associated with cell type proportions. Array batch and PlaNET-derived ancestry were also weakly associated with PC1 and PC2 in the processed dataset (p values < 0.001, R^2^ < 0.25). The fact that PC1 was more strongly associated with cohort than any other variable suggests that there are unidentified technical and/or biological variables contributing to between-cohort variability. In summary, data normalization and probe filtering are essential for reducing DNAme variance associated with technical factors, and cohort, array batch, cell type proportion, self-reported ethnicity, ancestry, and sex are all important variables to consider in any downstream analyses of these data.

### Placental genetic ancestry epiphenotype accurately captures SNP genotype-estimated ancestry

**Figure 2:**
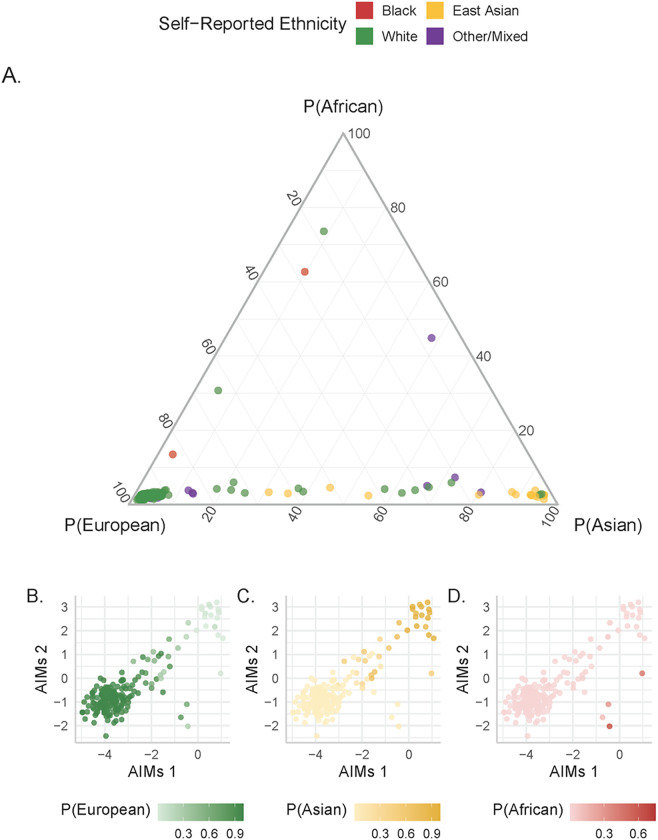
Relationship between PlaNET ancestry probabilities, self-reported maternal ethnicity, and Ancestry Informative Marker (AIMs) Coordinates. (A) Ternary plot of PlaNET ancestry probabilities (P(European), P(Asian) and P(African), colored by self-reported maternal ethnicity (Black, East Asian, white, other/mixed). Samples of unknown ethnicity were excluded. The three axis labels give 0 to 100 percent probabilities for samples belonging to each of the three ancestry groups. (B-D) Scatterplot of AIMs coordinates 1 (x-axis) and 2 (y-axis) colored by PlaNET ancestry probability score represented as a color gradient from 0 to 100 (Green for P(European) (B), Yellow for P(Asian) (C), and Red for P(African) (D)). Samples of European ancestry tend to cluster in the lower left; Asian in the upper right, and African in the lower right by AIMs coordinates.

DNAme variation is greatly influenced by genetic variation, which differs by ancestry of the individual. However, genetic ancestry data are often not measured, and while many pregnancy studies collect maternal self-reported ethnicity as an alternative measurement, this approach is inherently limited. First, ethnicity is a social concept that can be related to but is fundamentally different from genetic ancestry. Further, if only maternal ethnicity is collected, it ignores the other parent’s contribution to the placental genome and epigenome (31). In addition, genetic ancestry is interesting to study in its own right, as it may independently drive DNAme variation and/or confound other interesting associations. Previously, we created a tool to estimate genetic ancestry from the DNAme data directly (which is implemented in the PlaNET R package (25)), and here we compare this ancestry estimate to (i) maternal self-reported ethnicity (for details on ethnicity categories see Methods) and (ii) genetic ancestry assessed using Ancestry Informative Markers (AIMs), an independent set of SNPs that were genotyped for each placenta (32) (Fig. 2).

Most placentas (n = 172/204) had a high estimated probability (score > 75%) of European ancestry and for most of these (n = 151/172, 88%) the maternal self-reported ethnicity was “white”/European descent. All 17 placentas with a high probability (score > 75%) of East Asian ancestry had maternal self-reported “Asian” ethnicity, as did 4 additional samples (n = 21/204). No cases had a probability score > 75% of African ancestry, but of the 2/204 cases with a probability score > 50% of African ancestry, the maternal self-reported ethnicity was “Black” in one case. While there is a strong relationship between PlaNET ancestry estimates and maternal self-reported ethnicity, importantly, 24 placental samples (14% of the combined cohort) did not have values > 75% in any one ancestry dimension. This suggests a high degree of genetic diversity, which is important to consider in downstream analyses and cannot be captured by assigning samples to discrete ethnicities or ancestry groups. Looking at demographic and DNAme-derived variable relationships, we found that as expected, across all cohorts the various ethnicity and ancestry measures were associated (R^2^ 0.22–0.95) (Supp Fig. 1).

Although both PlaNET- and AIMs-inferred ancestry metrics yield continuous values in multiple dimensions of ancestry variation, the outputs of the two methods are not directly comparable. The two primary AIMs coordinates are sufficient to separate European, East Asian, and African samples, and were thus compared to the three PlaNET-derived ancestry probabilities (Fig. 2). In general, AIMS coordinates were found to correspond very well to PLANET ancestry probability scores, and most placentas that had values < 75% in the three PlaNET probabilities had AIMs scores in the first two coordinates in the mid-range of values. As few cases had high estimates for African ancestry by either method, we could not assess this ancestry dimension for interaction with other variables in subsequent analyses. As PlaNET ancestry probability is based on placental Illumina array data directly, our findings suggest that it is a useful tool for considering genetic variation that influences DNAme variation, particularly when matched genotyping data is not available.

### The placental epigenetic clock can predict reported gestational age in term placentas

Gestational age at birth is often unavailable in public datasets, but this variable is important to account for in placental studies as DNAme changes dramatically with gestational age, even late in pregnancy(33). Further, clinically-reported gestational age, which is estimated by first trimester ultrasound (gold standard), later ultrasound, or based on self-report of last menstrual period (LMP), is associated with inherent variability(34, 35). To address both of these problems, gestational age can be predicted from DNAme data itself, using several methods. Here, we applied the refined robust placental clock (RRPC) as it was developed to estimate gestational age specifically for term placentas, which make up the vast majority of our cohorts(33).

**Figure 3:**
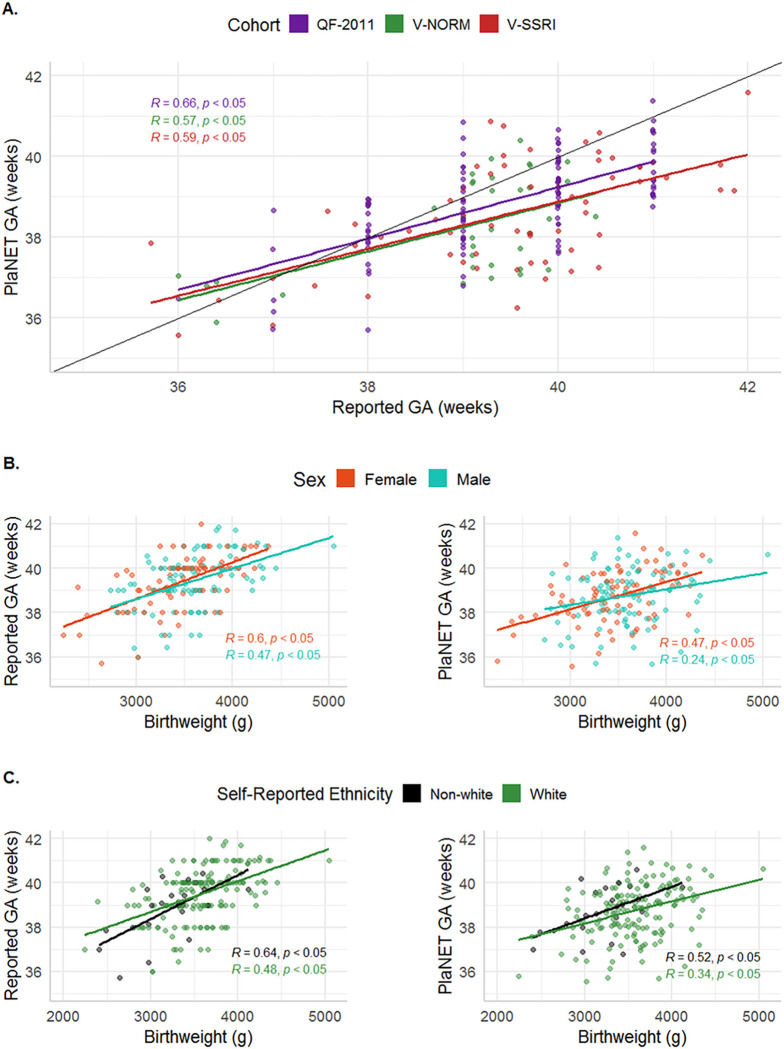
Relationships between PlaNET-estimated gestational age, clinically-reported gestational age, sex, birth weight, and self-reported ethnicity. (A) PlaNET-estimated gestational age (GA) using the refined-robust placental clock, compared to clinically-reported gestational age.(B) Reported and PlaNET-estimated GA versus birth weight, separated by sex.(C) Reported and PlaNET-estimated GA versus birth weight, separated by self-reported ethnicity.

In each of the three cohorts, we observed a moderate correlation between reported and estimated gestational age (Pearson’s R = 0.54, 0.59, and 0.66 for V-NORM, V-SSRI, and QF2011, respectively). The median deviations between predicted and reported gestational age were < 1 week in all three cohorts (median deviations − 0.51, −0.87, and − 0.57 weeks for V-NORM, V-SSRI, and QF2011, respectively) (Fig. 3). Considering the three cohorts together, the average median deviation between the RRPC and the reported gestational age was − 0.6 weeks, or −4.3 days. To contextualize this value, a study of > 500,000 pregnant individuals in California reported that LMP-based GA had an absolute deviation > 14 days in 17.2% of cases compared to ultrasound-derived GA (35), underscoring the relative accuracy of DNAme-based gestational age.

To further evaluate the gestational age epiphenotype, we compared both reported gestational age and RRPC-estimated gestational age to birth weight, as we expected both measurements of gestational age to correlate with infant size. Overall, clinically-reported gestational age was more strongly correlated with birth weight than was the RRPC-estimated gestational age (Pearson’s R of 0.54 and 0.37, respectively) (Fig. 3). We found that these gestational age-birth weight relationships were not significantly different by sex or maternal ethnicity (white vs. non-white), although both measures of gestational age tended to be more strongly correlated with birth weight in placentas that were female and of non-white maternal ethnicity (Fig. 3). These results suggest that the RRPC-predicted gestational age is less accurate than clinically-reported gestational age, at least in these cohorts, in which the range of gestational age at birth was small. However, these results also indicate that the RRPC is still quite accurate and could be used to predict gestational age when such data are missing, or when comparing gestational age across datasets, which may have different standards of gestational age estimation or reporting.

### Cell composition epiphenotype estimates can identify systematic differences between cohorts

The cell composition can vary between chorionic villi (bulk tissue) samples due to localized heterogeneity within the placenta, or due to systematic differences in sampling techniques between cohorts. As DNAme profiles vary markedly between cell types, they can be used to estimate the relative cell type proportions in whole chorionic villi samples (i.e., bulk tissue). Cell type proportions can then be compared between cohorts, datasets, or disease status groups to identify systematic between-group differences.

In assessing the inter-relationships between the different cell type proportions calculated with PlaNET, we found that cytotrophoblast proportion was inversely correlated with syncytiotrophoblast proportion, and that there were no strong relationships between cytotrophoblasts or syncytiotrophoblasts and any other cell type proportions, (Supp Fig. 1). The estimated proportions of Hofbauer cells and nRBCs, which are both typically very small, were also unrelated to other cell proportions.

The estimated distribution of major placental cell types was found to be similar across all three cohorts in our study (**Error! Reference source not found.**). We observed that the total proportion of trophoblasts (sum of syncytiotrophoblast and cytotrophoblast proportions) contributed to an average of 80.9% of each chorionic villus sample (SD = 3.6%; range 66.9–91.9%), while nucleated red blood cells (nRBCs) were present in only minor amounts (range 0.0–2.4%). The high trophoblast and low nRBC proportions confirm that, in these three cohorts, fetal blood contamination is negligible, and samples originate predominantly from the intermediate and terminal chorionic villi (15).

**Figure 4:**
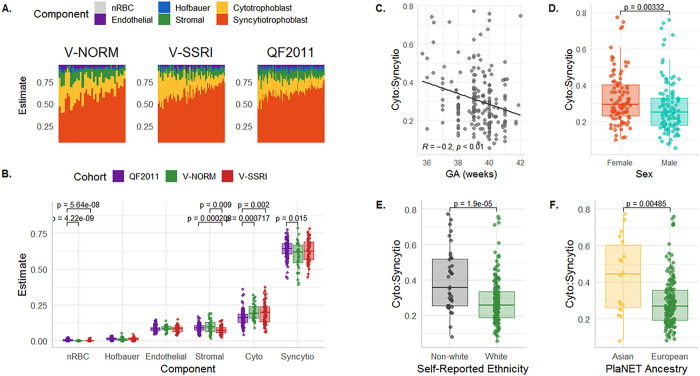
Association of cell type proportions with demographic variables. (A) PlaNET cell composition estimates across cohorts, shown as the estimated cell composition of each sample (column) by cohort. (B) The mean estimated proportion of each cell type separated by cohort.(C) Cytotrophoblast:syncytiotrophoblast ratio versus reported gestational age. (D) Cytotrophoblast:syncytiotrophoblast ratio by sex, (E) Self-reported white ethnicity, and (F) PlaNET European or Asian ancestry probability > 0.75.

When comparing relative cell proportions across cohorts, some subtle but significant differences were noted. A slight decrease in stromal cells was observed in V-SSRI samples as compared to the other two cohorts (Fig. 4B). As the V-SSRI cohort was an outlier in that it contained multiple samples with very long processing times (> 100 hours), we sought to evaluate whether processing time was associated with cell composition estimates (Supp Fig. 2). Increased processing time correlated with a reduction in stromal cell proportions, even when the SSRI dataset was removed to assess dataset-processing time confounds (R=−0.37, p < 0.05). Beyond this small impact on stromal cells, however, processing time appeared to have little effect on placental cell composition estimates.

Samples from the QF2011 cohort displayed a slightly higher median estimate of syncytiotrophoblasts, and a lower median estimate of cytotrophoblasts, as compared to the other two cohorts (Fig. 4B), leading to a lower cytotrophoblast:syncytiotrophoblast ratio. We therefore sought to evaluate whether any demographic variables might also be associated with cytotrophoblast:syncytiotrophoblast ratio, and observed a lower ratio in association with increasing gestational age, male sex, white maternal ethnicity, and European PlaNET ancestry probability > 75% (Fig. 4C–F). To distinguish the impact of ancestry/ethnicity and fetal sex on cell types, as opposed to possible cohort effects (as the QF2011 cohort mainly included mothers that self-reported as white and placentas with European predicted ancestry) we investigated the associations between cytotrophoblast:syncytiotrophoblast ratio and maternal white ethnicity and fetal sex in the V-SSRI and V-NORM cohorts separately. Considering only these two cohorts, we found no association between sex and estimated cell proportions, however, lower cytotrophoblast:syncytiotrophoblast ratio remained associated with both maternal white ethnicity and with a high (> 75%) European ancestry probability (Supp Fig. 3). Because ethnicity has been reported to potentially associate with gestational age at birth (36), we hypothesized that the observed trophoblast ratio/ethnicity relationship may be arising from a confounding association between trophoblast ratio and gestational age. In these cohorts, we did observe a slightly lower gestational age at birth in cases of non-white versus white maternal ethnicity (p = 0.0012), and also observed a decrease in cytotrophoblast:syncytiotrophoblast ratio with increasing gestational age (p = 0.00052). However, when the linear model testing for association between white/non-white ethnicity and cytotrophoblast:syncytiotrophoblast ratio was adjusted for gestational age, trophoblast ratio remained associated with ethnicity (higher ratio in non-white ethnicity) (p = 3.18e-7).

### Further evaluation of relationships between epiphenotypes and biological and technical variables

Before performing statistical analysis, it is important to assess inter-relationships and possible collinearities between demographic and technical variables in a dataset, including any relevant epiphenotype variables, and any necessarily related demographic variables, such as birth weight and gestational age. As the datasets originally used to construct the PlaNET epiphenotyping tools may have inherent biases towards different technical or biological variables, investigating the relationships between these epiphenotypes and other dataset metrics in these three well-characterized cohorts could provide useful knowledge for future applications of these tools. Reassuringly, beyond the factors already discussed, we did not detect further associations between PlaNET epivariables and other biological or technical variables (Supp Fig. 1).

Of the remaining variables of interest, birth weight standard deviation (SD) and placental efficiency (residual of fetal weight regressed on placental weight, sex, and gestational age) are both metrics of fetal growth during gestation, and are interesting to evaluate relative to the PlaNET tools for their relationships to both successful gestation and pathologic conditions such as preeclampsia or fetal growth restriction. Birthweight Z-score characterizes fetal growth by contextualizing infant birthweight relative to population-based reference groups of sex- and gestational-age-matched peers, while placental efficiency is a metric reflects the growth (mass) of a fetus relative to the growth (mass) of its own placenta. In principle, larger placentas can support larger infants, but the most “efficient” placentas are those that support adequate fetal growth with less relative placental mass. Birthweight Z score and placental efficiency were significantly associated with each other, but were not strongly associated with other variables (Supp Fig. 1). It is worth noting, however, that birth weight Z-score and gestational age were both higher in placentas with high PlaNET European ancestry probability score (p < 0.001; p < 0.01 respectively), and in cases with maternal white ethnicity (p < 0.01; p < 0.001). We found that fetal:placental weight ratio was higher at lower gestational ages as reported in (37), and this ratio was thus also associated with altered cell type proportions (Supp Fig. 4). The residual of fetal weight regressed on placental weight, as an improved measure of placental efficiency, however, was not associated with either gestational age or cell type composition (Supp Fig. 4). Beyond the factors already discussed, we did not detect further associations between PlaNET epivariables and other biological or technical variables.

## Discussion

**Figure 5:**
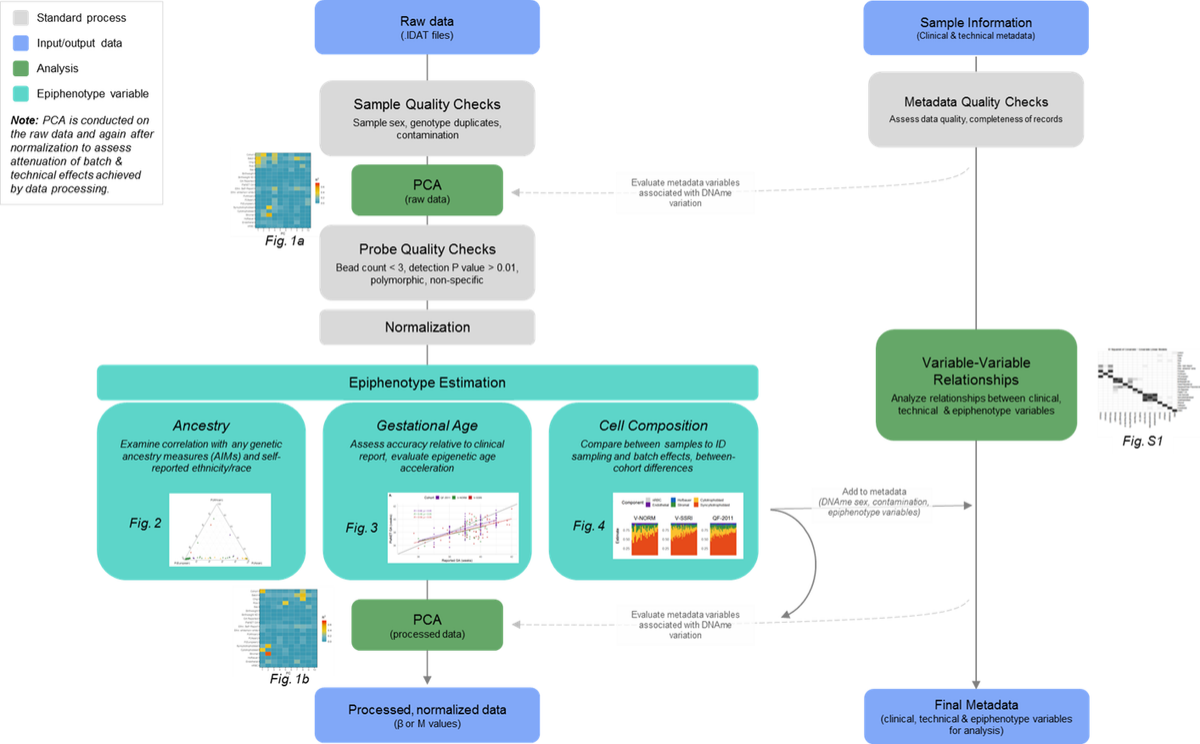
Suggested integration of placental epiphenotype variable estimation and analysis into DNAme processing and analysis pipelines.

Evaluation of the epiphenotyping tools assessed here indicates that they are appropriate for use in placental DNAme data processing and analysis in a variety of contexts, and we therefore recommend their regular integration on in standard processing and analysis pipelines for placental DNAme data. Our major findings are presented in Table 2. In summary, we find that these placental epiphenotype variables first enable detailed technical assessment of data quality, such as the fact that inter-centre and sampling differences can be identified by comparing cell composition across samples). Additionally, epiphenotypes enable analysts to evaluate metadata reporting accuracy by comparing each epiphenotype variable to analogous clinically-reported data, such as by comparing PlaNET ancestry to genetic ancestry (AIMs), or by comparing epigenetic age to reported gestational age. See Fig. 5 for an overview of the suggested integration of epiphenotype estimation in a DNAme processing pipeline.

PlaNET can first be used to estimate ancestry along three continuous axes of variation. The main utility of these PlaNET ancestry probabilities is that they can account for ancestry-driven genetic variation that influences DNAme, particularly when genetic ancestry data are absent. Self-reported ethnicity is often collected and used as an estimate genetic ancestry, but even when both maternal and paternal ethnicity data are available for prenatal samples such as placenta, ethnicity is a categorical identity and a poor proxy for genetic ancestry, which is continuous in nature and more closely reflects genetic variation(38–40). While human populations are much more diverse than can be captured on the three dimensions predicted by this tool (African, East-Asian, European)(38), it is a useful method for ancestry estimation when independent genetic data are unavailable. As DNAme data from more diverse populations becomes available, new tools can and should be created that improve upon the current ability to distinguish diverse genetic ancestries. It is also important to note that PlaNET or genetic ancestry estimates are not a substitute for self-reported ethnicity, which may be associated with important social determinants of health(40), including lifestyle and exposure factors interacting with the *in-utero* environment. It is difficult to examine the effects of self-reported ethnicity independently from genetic ancestry, as the two are typically associated, though in this dataset, maternal ethnicity showed a slightly stronger association with PC1 (largest proportion of DNAme variation in the processed data) than did estimated ancestry. Both ancestry and ethnicity or race should be considered in DNAme analyses when applicable; many methylated sites are strongly associated with nearby genetic variants (41), and environmental effects (which may be captured by self-reported ethnicity) should be examined in the context of this underlying genetic variation.

The PlaNET placental gestational age clock (RRPC) was less strongly correlated with birth weight than was clinically-reported gestational age, which implies reduced accuracy of the DNAme-derived estimate. Nonetheless, the RRPC gestational age on average deviated only by −4.3 days from the reported gestational age, which is less than reported in the original publication of this tool (r = 0.26 with an absolute mean deviation of 7 days) (18). The Lee et al. (2019) study utilized publicly available placental DNAme datasets, and it is possible these were subject to variable quality of clinical records, which may explain the higher accuracy observed in our samples. While the present study focused on evaluating tools presented in the PlaNET R package, two other placental gestational age clocks, exist, developed by Mayne et al. (2017) (42) and Haftorn et al. (2021) (43). The first was trained on publicly available placental DNAme datasets of diverse pathologies and has a median absolute deviation of (predicted – reported age) ± 1.47 weeks, while the Haftorn et al. clock was trained on placental samples from a well-characterized Finnish cohort, and had a mean absolute deviation of ± 3.6 days, which was similar to what we observed in the present study with the RRPC.

Regarding cell composition, the placenta is a heterogenous solid tissue with multiple cell types, derived from all three components of the blastocyst: 1) trophoblast from trophectoderm; 2) placental endothelial and endodermal cells from hypoblast(44, 45), and 3) Hofbauer cells from epiblast(15). Each cell type in the placenta has a unique DNAme signature, which contributes to DNAme differences across samples of bulk chorionic villi(15). Cell type composition differences are known to be a major source of variation in DNAme data in general, beyond just the placenta(46). Although we could not validate our cell type proportion estimates (as the bulk of nuclei come from the multinucleated syncytiotrophoblast and accurate counts are not possible), the ratios observed here were consistent with our sampling technique, which aims to obtain consistent samples of free floating intermediate and terminal villi free of very large vessels and washed well of any contaminating blood(47, 48). Using the PlaNET cell proportions has also been shown to be superior to reference-free approaches applied to the placenta(49). Overall, based on cell proportions, placental sampling technique applied here appears to have been consistent between QF2011 (sampled in Brisbane, Australia) and the two Vancouver cohorts: V-NORM and V-SSRI. This between-cohort consistency in cell composition is reassuring, although in other studies we have observed large variations in cell composition between public datasets(15). We thus suggest that when estimated placental cell composition indicates that total trophoblast proportions are significantly beyond the range of ~ 0.65–0.92, as seen here in our three representative cohorts, studies may benefit from sample removal to ensure homogeneous study groups, or the region of the placenta/method of sampling should be considered for possible interaction with results. Specifically, we anticipate that the proportions of total trophoblast, endothelial cells, and stromal cells in a sample may be related to sampling technique. For example, we have observed that trophoblast levels are lower if placental chorionic villi are sampled closer to the fetal facing surface of the organ (immediately under the chorionic membrane), where larger vessels reside (stem villi) (15).

The cytotrophoblast:syncytiotrophoblast ratio was found to be strongly associated with gestational age over the last few weeks of gestation, which is consistent with the decrease of cytotrophoblast populations over time as these cells fuse to form the multinucleated syncytiotrophoblast, which in turn becomes increasingly abundant towards full term (15, 50). The association between cytotrophoblast:syncytiotrophoblast ratio and maternal ethnicity/placental genetic ancestry (lower ratio in non-white and in low probability European placentas) can be partly explained by a reduced gestational age in these cases; however, given the association observed in our data between non-white ethnicity and reduced birth weight standard deviation (even when accounting for gestational age), socio-cultural influences may also be at play and should be explored in more depth in future studies. As placental pathology or environmental exposures may be associated with altered cell composition, which in turn contributes to changes in DNAme in bulk tissue, analysts should carefully consider how and when to correct for cell type composition in bulk tissues epigenetic analyses.

The interrelationships between variables that affect placental DNAme are important to understand before undertaking further analysis. While we identified a small number of variables that differed between cohorts, including differences in ancestry composition and slight differences in trophoblast ratios, none of these factors individually explained as much variation in DNAme as the “cohort” variable itself did (significantly associated with PC1). This is an expected result, and cohort-level differences likely arise from the combination of many factors including different procedures used in sample processing, storage and DNA extraction, and differences in environmental exposure between samples comprising each cohort (e.g. diet, medication, environmental exposure, stress). This is particularly relevant as in this study, the QF2011 cohort was exposed to an acute environmental stressor (flood), which will be explored in a future study for its effect on DNAme. Although a subset of the V-SSRI cohort was exposed to SSRIs and gestational maternal depression, in a previous study we found no consistent signature of altered placental DNAme in association with these exposures, and thus this particular exposure variable is likely not a large driver of cohort-level differences(29).

Obstetrical outcomes can differ by the sex of the conceptus; for example, male (XY) placentas tend to be larger and more prone to proinflammatory response than female (XX) placentas (51). Accordingly, we explored whether any epiphenotype variables were associated with sex, and found that overall sex was not strongly associated with ancestry, gestational age, or cell composition epiphenotype variables. Sex was also not associated with the first two principal components of DNAme variation in these three cohorts. Interestingly, a slightly higher cytotrophoblast: syncytiotrophoblast ratio was observed in female samples, but this effect was limited to the QF cohort (Supp Fig. 3). We did not observe sex differences in placental cell proportions in a previous study with a combined cohort size of n = 343(16), implying that the observed higher female cytotrophoblast:syncytiotrophoblast ratio finding could either be due to statistical noise in these cohorts, or be related to unmeasured cohort-specific factors. Sex differences in DNAme (16, 52, 53) and gene expression profiles (54) have been observed at autosomal loci in the placenta, likely secondary to sex chromosome-related gene expression sex differences(16). Additionally, a recent study indicated that placental DNAme patterns associated with gestational age may be driven by changes in cell composition across gestation, and suggested that these changes in cell composition across gestation may also differ between male and female placentas, although effect sizes were small(55). Thus, if estimated placental cell composition and gestational age do vary by sex, this variation is likely of small effect size.

In summary, cohort, array batch, cell type proportions, self-reported ethnicity, genetic ancestry, and biological sex are important variables to consider in any analyses of Illumina DNAme data. We find that estimating epiphenotype variables (gestational age, ancestry, cell proportions) from the DNAme itself, when possible, provides both an independent check of clinically-obtained data and can provide a robust approach to compare variables across different datasets. The method by which technical, biological and epiphenotype variables are accounted for in analyses should be carefully considered, given the associations observed here between cell composition, ethnicity, genetic ancestry and gestational age/birth weight Z-score. Adjusting or “controlling” for these factors in statistical models can mask important relationships between these variables and the outcomes of interest. As factors such as cell composition, ethnicity, genetic ancestry, and gestational age/birth weight may all interact with fetal health in unique ways, they should be studied directly when possible. If sample size is sufficient, for example, data should be analyzed separately by maternal self-reported ethnicity groups, and by sex, since DNAme alterations associated with other variables of interest may differ within these groups. Though not explored here, it is also worth noting that epiphenotypes could be used for metadata harmonization across cohorts with different reporting standards (one could calculate the epigenetic gestational age for all samples and using these values in downstream analysis). We also note that these epiphenotype variables can be analyzed directly in relation to outcome variables of interest, such as disease status (e.g. Are cell type proportions altered in disease contexts? Does epigenetic age increase relative to reported gestational age in disease contexts?).

Overall, we recommend the application of epiphenotyping approaches, followed by detailed exploration the interrelated nature of biological, technical, and epiphenotype variables in any dataset before beginning analysis, and further recommend that analysts exercise due caution in interpreting results.

## Materials and Methods

### Cohorts

204 placentas from three cohorts were processed for DNAme arrays in Vancouver, Canada. The three cohorts consisted of: (i) V-NORM, a normative population of pregnancies recruited at BC Women’s Hospital (BCWH) in Vancouver, Canada (n = 35), as part of a study on Epigenetics in Pregnancy Complications (EPIC)(7, 26, 27); ii) V-SSRI, a population of pregnant individuals recruited in Vancouver, Canada in the 20th week of gestation (n = 64), with/without clinical depression, and with/without the use of selective serotonin reuptake inhibitors (SSRIs) (28, 29); and (iii) QF2011, a population of pregnancies exposed to a sudden-onset disaster during gestation due to catastrophic flooding in the Australian state of Queensland in early January 2011 (n = 105)(30). Ethics approval for the V-NORM and V-SSRI cohorts, as well as overall approval for the present study was obtained by the University of British Columbia/Children’s and Women’s Health Centre of British Columbia Research Ethics Board (H04–70488, H12–00733, and H16–02280 respectively). The QF2011 study received ethics approval for the initial and follow-up protocols from the Mater Hospital Human Research Ethics Committee (1709M, 1844M). The QF2011 study also has ethics approval from the University of Queensland Human Research Ethics Committee (2013001236). For all cohorts, written informed consent was obtained from all participants, and all procedures complied with the ethical standards on human experimentation and with the Helsinki Declaration of 1975 (revised in 2008). A subset of V-NORM participants were recruited by the BC Children’s Hospital BioBank (BCCHB) (Vancouver, BC) an institutional biobank that collects samples and data from both children and women at BC Children’s and Women’s Hospitals and Health Centres for future, ethically-approved research.

The V-SSRI and QF2011 were prospectively recruited cohorts, and gestational ages ranged from 36 weeks to term. Cases for V-NORM were thus limited to gestational ages at birth > 36 weeks to match the other two cohorts. Exclusion criteria applied to all cohorts included pregnancies with multiple fetuses or chromosome abnormalities. Additionally, V-SSRI excluded mothers with bipolar illnesses, hypertension, current substance abuse, any diabetes, or infants with congenital brain malformations or fetal growth. V-NORM excluded any pregnancies affected by preeclampsia, while QF2011 was not subject to any additional specific exclusions. Any exclusion criteria applied were intended to minimize the presence of confounding factors known to associate with DNAme alterations (such as chromosome abnormalities or preeclampsia) or outcome variables of interest (e.g. bipolar illness possibly confounding depression analyses in V-SSRI).

Self-reported ethnicity and/or race are increasingly recognized as important variables to consider in health research, but there have not been consensus definitions of race or ethnicity (56–58). Further, socially meaningful groupings may differ across countries and cultures, or even change for an individual over time (56). To harmonize these self-reported variables across cohorts, as well as to create groups with sufficient sample size for analysis, we have chosen to group samples by maternal self-declared race/ethnicity as follows: (i) “white” if reported as white, Caucasian, European, or from any European country; (ii)“Asian” if reported as Asian, Chinese, Japanese, Korean, Filipino, Vietnamese or Thai; (iii)“Black” if reported as Black or African; (iv) and “Other” if reported as Pacific Islander, South Asian, South American, Middle Eastern, Latin American, any specific country within those areas, or mixed ethnicity. We acknowledge, however, that these are imperfect descriptors, and that these groupings may not accurately reflect the intended response of the participants.

Infant birth weight is presented as standard deviation Z-scores from the mean sex- and gestational age-specific birth weights, based on Canadian birth charts(59). Placental efficiency was calculated as the residual of birth weight regressed on placental weight, adjusted for gestational age and sex(37). This residual is independent of gestational age, whereas infant birth weight to placental weight ratio is positively correlated with gestational age(37). Untrimmed placental weight (placental weight including the reflected amniotic and chorionic membranes), rather than trimmed weight, was used for placental efficiency calculations as it was available in a greater number of cases, and the trimmed and untrimmed values were highly correlated in cases for which both measurements were available (n = 75, Spearman’s Rho = 0.97, p < 2.2e-16). Between-cohort differences were evaluated by ANOVA for continuous variables and Chi-square tests for categorical variables.

### Placental Sampling

Placental sampling after delivery followed two similar but distinct sampling processes. First, the V-NORM and V-SSRI cohorts were sampled by a single lab in Vancouver, Canada using a standardized sampling protocol(27). Briefly, 1.5–2 cm^3^ samples of chorionic villi were taken from each of four distinct cotyledons (sites) from below the surface of the fetal-facing side of the placental disc at a depth that targeted intermediate and tertiary villi. Placental processing time (number of hours from placenta delivery until sampling) ranged from 0.5 hours to 288 hours (with 5 samples missing data). The samples were washed thoroughly to remove blood, and any thick vessels were removed. Samples were frozen at −20°C until use. DNA was then extracted from all four cotyledon samples using a salting-out DNA extraction procedure(60), and extracted DNA from the four sites was pooled in equimolar proportions to provide a representative sample of each placenta. The second sampling process involved the QF2011 placentas, which were processed in Brisbane, Australia within 60 minutes of delivery, and eight sites (1 cm^3^ each) representing different cotyledons were sampled across the fetal-facing side of each placenta. These samples were snap frozen in liquid nitrogen and subsequently shipped to Montreal, Canada. Pools of five samples were ground over dry ice, and DNA was extracted using the DNeasy Blood & Tissue Kit (Qiagen, Valencia, CA, USA) in Montreal before being shipped to Vancouver on dry ice for DNAme processing.

### DNAme arrays and data quality checks

DNA samples from all three cohorts were run on Illumina Infinium MethylationEPIC arrays in Vancouver, BC, Canada. Processing included DNA purification after extraction using the DNeasy Blood & Tissue Kit (Qiagen, Valencia, CA, USA), bisulfite conversion using the EZ DNAme Kit (Zymo Research, Orange, CA, USA), and hybridization to and processing of the Illumina Infinium MethylationEPIC BeadChip arrays according to the manufacturer’s protocol (Illumina, San Diego, CA, USA). Samples from the three cohorts were distributed and run in 3 array batches across 44 eight-sample chips as illustrated in Supp Fig. 5. Samples were carefully distributed across array chips (1–44) and rows (1–8) with respect to the following variables, to minimize potential batch effects: exposure groups (SSRI exposed/non-exposed and QF2011 objective flood stress high/low) and infant sex (all cohorts).

DNAme data from raw IDAT files were read into R v4.2.2(61) and annotated with the Illumina Infinium MethylationEPIC v1.0 B4 Manifest. Several data quality control checks were undertaken using the R packages minfi (62, 63), wateRmelon(64, 65), and ewastools(66). First, each sample was assessed at 17 Illumina control probes to evaluate bisulfite conversion efficiency and array run quality; all samples passed the manufacturer-recommended thresholds at the control probes. Next, average total (methylated + unmethylated) fluorescence intensity was assessed between samples, and between array batches. All samples had similar total fluorescence, though samples run on the EPIC array in Batch 3 had slightly higher average intensities than those in Batch 1 and 2. Sample sex was assessed with the ewastools package(66), using the mean total fluorescence intensity (methylated + unmethylated) of the X and Y chromosome probes, normalized to the per-sample mean autosomal total fluorescence intensity, and was confirmed to match the clinically-reported sex of the infant in all cases. Sample genetic identity was assessed using the 59 SNP (‘rs’) probes on the EPIC array with the “call_genotypes” and “enumerate_sample_donors” functions (*ewastools*)(66). Finally, DNAme beta value density plots of all samples were visually assessed to determine overall similarity of the beta value distributions between samples, with no outliers identified.

### Epiphenotype estimation

The PlaNET R package(25) was used to determine DNAme-based estimates of genetic ancestry, placental cell type composition, and gestational age at birth. These metrics were calculated based on BMIQ-noob normalized data before probe filtering, as recommended in the PlaNET package documentation(25). *PlaNET-derived genetic ancestry* can be described as a continuous variable on three axes of variation that sum to one, representing contributions of African, East-Asian, and European ancestry(24). *PlaNET-derived cell composition* was calculated using the robust partial corrections method, which yields six compositional estimates of the major placental cell types (endothelial cells, stromal cells, Hofbauer cells, nucleated red blood cells, cytotrophoblasts, and syncytiotrophoblasts) (15). To avoid confusion, we use the term “Cytotrophoblast” for the cell type PlaNET reports as “Trophoblasts”, as these were single-nuclear trophoblasts derived from chorionic villi, and represent stem and columnar cytotrophoblast, but would not be expected to have significant contribution from extra-villous trophoblast or syncytiotrophoblast(15). *PlaNET-derived gestational age* can be calculated using 3 different built-in tools: the robust placental clock (RPC), the control placental clock (CPC), and the refined robust placental clock (RRPC). The RRPC is most appropriate to the present dataset as it was developed using exclusively samples > 36 weeks of gestational age (including pathological samples) to improve prediction over the narrow age range at term(18).

### Data processing

After estimation of epiphenotype variables, raw data were normalized for analysis, using noob and dasen combined normalization methods (67, 68). This method was found to outperform functional, BMIQ, SWAN, and quantile normalization, all with and without noob where possible (69–73), based on the increased correlation of technical replicate pairs after normalization and amelioration of probe type bias. Correction of probe type bias was evaluated quantitatively by comparing the maxima and minima of Type I versus Type II probes before and after normalization (Khan et al., in prep). Subsequently, poor-quality probes (detection p value > 0.01 or bead count < 3 or missing values in > 5% of samples) were removed from the dataset (n = 4,783), as were cross-hybridizing probes and probes overlapping single nucleotide polymorphisms (MASK_general column of (74), n = 99,360). Technical replicates of twelve genetically-distinct samples (11 replicate pairs and one sample run in quintuplicate) were used to assess data processing by calculating the correlation between all DNAme beta values of replicate sample pairs in the raw and processed datasets. The highest quality replicate from each pair was retained for the rest of the analysis, and all others removed (n = 15 replicate samples removed). One additional non-replicate sample was removed for failing probe quality checks (> 1% of array probes failed detection P/bead count). After data processing and quality control, a total of 746,608 probes in 204 samples remained for analysis.

### Principal component analysis

*Principal component analysis (PCA)* was used to assess the primary drivers of DNAme variance in the data using the R package irlba(75). Linear models were run to assess covariance between each principal component and technical and biological variables (PC ~ dependent variable) using the plomics package(76), and visualized in a heatmap method similar to (77).

### AIMs data processing

*Ancestry informative markers (AIMS)* were used as an independent assessment of genetic ancestry. Genotypes at 57 single-nucleotide polymorphisms informative to assess African, East Asian, and European ancestry(32, 78) were obtained using the Sequenom iPlexGold assay for 192/204 samples, and analyzed as previously described(32). Briefly, for each sample individually, AIMS data were combined with external data from 2,418 individuals from the 1000 Genomes Project (1KGP), serving as ancestry reference populations. Multidimensional scaling (MDS) was then run on the Euclidean distance matrix based on genotype of these samples (coded numerically by the B allele frequency as 0, 1, or 2). The top two MDS coordinates were used to describe ancestry for each sample across a continuum relative to 1KGP samples of East-Asian, African, and European ancestry, and are denoted throughout the article as AIMs coordinates.

## Figures and Tables

**Table 1:** Summary of key biological and technical variables for each cohort. SD refers to standard deviation, QC refers to quality control.

	V-NORM	V-SSRI	QF2011	p value*
n	35	64	105	
**Biological variables**				
Gestational age at birth (mean weeks (SD))	39.0 (1.1)	39.47 (1.3)	39.4 (1.2)	0.29
Infant sex (n male (%))	19 (54.3)	33 (51.6)	59 (56.2)	0.84
Infant birthweight (mean grams (SD))	3412.5 (537.8)	3451.7 (460.7)	3584.2 (403.4)	0.06
Infant birthweight (mean Z-score (SD))	−0.02 (1.08)	−0.02 (0.81)	0.28 (0.82)	0.04
Placental efficiency	−0.09 (1.1)	−0.10 (0.93)	0.10 (0.99)	0.37
Population	British Columbia, Canada	British Columbia, Canada	Queensland, Australia	
*Self-Reported Ethnicity (n (%))*				< 0.001
White	14 (40.0)	48 (75.0)	102 (97.1)	
Asian	12 (34.3)	6 (9.4)	1 (1.0)	
Black	0	1 (1.6)	1 (1.0)	
Other	8 (22.9)	1 (1.6)	0 (0.0)	
Missing	1 (2.9)	1 (1.6)	0 (0.0)	
**Technical variables**				
Processing time (median hours (SD))	2.9 (18.9)	28.7 (59.8)	Approximately 1 hour	
Storage	−20C	−20C	Snap Frozen Liquid N2	
DNA extraction method, location	Salt extraction, Vancouver	Salt extraction, Vancouver	Qiagen DNeasy Blood and Tissue kit, Montreal, Canada	
Number of technical replicates	4	0	8	
*EPIC Batch Distribution (n (%))*				
Batch 1	0 (0.0)	64 (100.0)	94 (89.5)	
Batch 2	18 (51.4)	0 (0.0)	0 (0.0)	
Batch 3	16 (45.7)	0 (0.0)	11 (10.5)	

* p values are based on ANOVA for continuous variables and Chi-square tests for categorical variables.

**Table 2:** Summary of findings in the evaluation of PlaNET package epiphenotyping tools for genetic ancestry, gestational age, and placental cell type composition.

	PlaNET Epiphenotype Variable		
	Ancestry	Gestational Age (refined robust clock, RRPC)	Cell composition
**Validation**	PlaNET ancestry is highly correlated with independent SNP genotype derived ancestry- informative markers (AIMs).	The refined robust placenta clock (RRPC) is less accurate than clinically reported gestational age for term placentas, but predicts gestational age on average within 4 days.	Cell composition results as expected for term chorionic villi: e.g. 70%-90% total trophoblast; <2% nucleated red blood cells.
**Calculation considerations**	PlaNET ancestry estimates are affected by normalization; data must be BMIQ + noob normalized prior to estimation(25).	Accuracy of placenta epigenetic clocks likely depends on data quality and uniformity of bulk tissue sampling.	Recommended to estimate cell composition on BMIQ + noob normalized data(25).
**Epiphenotype variation by:**			
**Cohort of origin**	QF2011 has predominant European ancestry, while V-NORM and V-SSRI are more diverse.	Similar across cohorts.	Altered trophoblast ratio in QF2011 cohort
**Processing time**	No association.	No association.	Slight decrease in stromal cell composition at high processing times
**Sampling Method**	Not evaluated.	Not evaluated.	Yes, previously reported(15), though not able to be evaluated in the present similarly-sampled cohorts
**Sex**	No association.	No association.	No association.
**Ethnicity**	Correlated with maternal ethnicity; but includes paternal contribution and is continuous not categorical.	No association	Possibly higher ratio of cytotrophoblast:syncytiotrophoblast in placentas with Asian ancestry.
	**PlaNET Epiphenotype Variable**		
**Reported Gestational Age**	N/A	Accelerated epigenetic gestational age is reported in preeclampsia(18), though this work was conducted with an earlier placental epigenetic clock (not the RRPC).	Decrease in cytotrophoblast:syncytiotrophoblast ratio with increasing gestational age.
**Cell Composition**	No association.	Decrease in the ratio of cytotrophoblast:syncytiotrophoblast with increasing gestational age, but RRPC gestational age is robust to cell composition.	N/A

## Data Availability

The data supporting the conclusions of this article are available in the Gene Expression Omnibus repository under accession GSE232778 in both raw and processed form. The raw data has been uploaded without inclusion of the EPIC ‘rs’ genotyping probes to protect participants’ genetic privacy, this information will be shared upon reasonable request to the authors.
